# Characterization of *Salmonella enterica* serovar Enteritidis isolates recovered from blood and stool specimens in Thailand

**DOI:** 10.1186/1471-2180-12-92

**Published:** 2012-06-06

**Authors:** Rene S Hendriksen, Eija Hyytia-Trees, Chaiwat Pulsrikarn, Srirat Pornruangwong, Phattharaporn Chaichana, Christina Aaby Svendsen, Rafiq Ahmed, Matthew Mikoleit

**Affiliations:** 1 Division of Bacterial Genomics and Epidemiology, WHO Collaborating Center for Antimicrobial Resistance in Food borne Pathogens and European Union Reference Laboratory for Antimicrobial Resistance, National Food Institute, Technical University of Denmark, Kemitorvet, Building 204, DK-2800, Kgs. Lyngby, Denmark; 2 Division of Foodborne, Waterborne, and Environmental Diseases; Enteric Diseases Laboratory Branch; Centers for Disease Control and Prevention, National Center for Emerging and Zoonotic Infectious Diseases, Mail stop CO3, 1600 Clifton Road, Atlanta, GA, 30333, USA; 3 Department of Medical Sciences, WHO National Salmonella and Shigella Center, National Institute of Health, Ministry of Public Health, Tiwanond Road, Amphur Muang, Nonthaburi, 11000, Thailand; 4 National Microbiology Laboratory, Public Health Agency of Canada, 1015 Arlington Street, Winnipeg, MB, R3E 3R2, Canada

## Abstract

**Background:**

Bacteremia due to *Salmonella* spp. is a life-threatening condition and is commonly associated with immune compromise. A 2009 observational study estimated risk factors for the ten most common non-typhoidal *Salmonella* (NTS) serovars isolated from Thai patients between 2002–2007. In this study, 60.8% of *Salmonella enterica* serovar Enteritidis isolates (n = 1517) were recovered from blood specimens and infection with *Salmonella* serovar Enteritidis was a statistically significant risk factor for bacteremia when compared to other NTS serovars. Based on this information, we characterized a subset of isolates collected in 2008 to determine if specific clones were recovered from blood or stool specimens at a higher rate. Twenty blood isolates and 20 stool isolates were selected for antimicrobial resistance testing (MIC), phage typing, PFGE, and MLVA.

**Result:**

Eight antibiogrammes, seven MLVA types, 14 *Xba*I/*Bln*I PFGE pattern combinations, and 11 phage types were observed indicating considerable diversity among the 40 isolates characterized. Composite analysis based on PFGE and MLVA data revealed 22 genotypes. Seven of the genotypes containing two or more isolates were from both stool and blood specimens originating from various months and zones. Additionally, those genotypes were all further discriminated by phage type and/or antibiogramme. Ninety percent of the isolates were ciprofloxacin resistant.

**Conclusions:**

The increased percentage of bloodstream infections as described in the 2009 observational study could not be attributed to a single clone. Future efforts should focus on assessing the immune status of bacteriaemic patients and identifying prevention and control measures, including attribution studies characterizing non-clinical (animal, food, and environmental) isolates.

## Background

*Salmonella enterica* is a common cause of human gastroenteritis and bacteremia worldwide [1-3] and a wide variety of animals, particularly food animals, have been identified as reservoirs for non-typhoidal *Salmonella*[4].

Although approximately 2,600 serovars of *Salmonella enterica* have been identified, most human infections are caused by a limited number of serovars and in general these infections are self-limiting [1]. However, approximately 5% of patients infected with non-typhoidal *Salmonella,* will develop bacteremia. The very young, elderly, and those with underlying disease are at a significantly higher risk for developing bacteremia when compared to patients with enteric salmonellosis. Bacteriaemic patients have higher rates of hospitalization, often have prolonged courses of illness and have higher case fatality rates [1,5].

Worldwide, *Salmonella enterica* serovars Enteritidis and Typhimurium are consistently ranked as the two serovars most frequently associated with human disease [6]. However, these rankings may considerably vary by geographic region and may change over time. A recent study showed that in 2007, *Salmonella* serovar Enteritidis accounted for 55% of all human *Salmonella* infections reported to the World Health Organization Global Foodborne Infections Network Country Data Bank [6]. In that same year, *Salmonella* serovar Enteritidis only accounted for 16% of human salmonellosis cases in Thailand [7].

In 2009, an observational study based on patient data from 11,656 *Salmonella* isolates collected between 2002 – 2007 estimated risk factors for the ten most common *Salmonella* serovars isolated from Thai patients [7]. In the study, 60.8% of *Salmonella* serovar Enteritidis isolates (n = 1517) were recovered from blood specimens and infection with *Salmonella* serovar Enteritidis was a statistically significant risk factor for bacteremia (odds ratio of 11.12; 95% CI 9.77 – 12.66) when compared to the other NTS serovars. In comparison, approximately 6% of *Salmonella* serovar Enteritidis isolates in the United States are recovered from blood (CDC unpublished data).

A previous study described an apparently invasive clone of a different *Salmonella* serovar in another region. However this study focused strictly on blood isolates [8]. For this study, we felt it would be important to characterize both blood and stool isolates. Characterization and comparison of blood and stool isolates is crucial for determining if there is a true increase in invasiveness or if patients are simply becoming infected with a regionally dominant clone.

The objective of this study was to characterize *Salmonella* serovar Enteritidis isolates causing human gastroenteritis and bacteremia in Thailand in a spatial and temporal context in order to determine if bloodstream infections are being caused by an invasive clone of *Salmonella* serovar Enteritidis. Isolates were characterized utilizing minimum inhibitory concentration (MIC) determination for antimicrobial resistance, phage typing, pulsed-field gel electrophoresis (PFGE), and Multiple-Locus Variable number tandem repeat Analysis (MLVA).

## Methods

### Bacterial isolates

The WHO National *Salmonella* and *Shigella* Centre in Nonthaburi receives all presumptive positive *Salmonella* isolates from all diagnostic laboratories throughout Thailand. In 2008, 444 isolates were identified as *Salmonella* serovar Enteritidis. Forty were selected for further study. Twenty isolates were recovered from blood specimens and 20 were recovered from stool specimens (fecal specimens or rectal swabs). Patient log-sheets were reviewed to insure that only one isolate per patient was included the study. Isolates were selected to insure geographic (Zones: 1, 3, 4, 10, 11, 12, & Bangkok BKK), age (5 month to 89 years), and seasonal (all isolates collected from January to December with exception of August) distribution. An equal number of stool and blood isolates were submitted from each zone.

### Serotyping

Isolates were serotyped using slide agglutination. O and H antigens were characterized by agglutination with hyperimmune sera (S & A reagents lab, Ltd, Bangkok, Thailand) and a serotype was assigned according to the Kauffmann-White scheme [9]. At CDC, the serotype was confirmed and PCR testing for the *Salmonella* serovar Enteritidis specific marker Sdf was performed [10].

### Antimicrobial susceptibility testing

MIC testing was performed at National Food Institute (DTU-Food) in Denmark using a commercially prepared, dehydrated panel, Sensititre, from TREK Diagnostic Systems Ltd. (East Grinstead, England). Antimicrobials and resistance cut-off values or clinical breakpoints used in the study were: ampicillin, AMP (R > 8 mg/L); amoxicillin + clavulanic acid, AMC (R ≥ 32 mg/L); apramycin, APR (R ≥ 32 mg/L); cefotaxime, CTX (R > 0.5 mg/L); ceftiofur, XNL (R > 2 mg/L); chloramphenicol, CHL (R > 16 mg/L); ciprofloxacin, CIP (R > 0.064 mg/L); colistin COL (R > 2 mg/L); florfenicol, FFN (R > 16 mg/L); gentamicin, GEN (R > 2 mg/L); nalidixic acid, NAL (R > 16 mg/L); neomycin, NEO (R > 4 mg/L); spectinomycin, SPT (R ≥ 64 mg/L); streptomycin, STR (R > 16 mg/L); sulphamethoxazole, SMX (R ≥ 256 mg/L); tetracycline, TET (R > 8 mg/L); and trimethoprim, TMP (R > 2 mg/L). Epidemiological cut-off values were interpreted according to current EUCAST (http://www.eucast.org) and European Food Safety Authority (EFSA) recommendations. Exceptions were made for interpretation of AMC, SMX, and SPT, where Clinical and Laboratory Standards Institute (CLSI) guidelines and clinical breakpoints were used [11-13]. Due to the absence of some epidemiological cut-off values in the EUCAST system and clinical breakpoints from CLSI, exceptions were made for the interpretation of APR MIC values which were interpreted according to research results from DTU. Quality control using *E. coli* ATCC 25922 was conducted according to CLSI [12,13].

### Phage typing

Phage typing was performed at the National Microbiology Laboratory, Public Health Agency of Canada, Winnipeg, MB, Canada using the Enteritidis phage typing scheme provided by the Health Protection Agency, Colindale, London, UK. This phage-typing scheme is composed of 17 *Salmonella* serovar Enteritidis specific phages. Isolates with lytic patterns that did not match standard phage lytic profiles were assigned an atypical phage type [14].

### Pulsed-field gel electrophoresis

PFGE was performed at DTU-Food using *Xba*I and *Bnl*I macrorestriction enzymes (Fermentas, Glen Burnie, Maryland, United States) according to the CDC PulseNet protocol [15]. The patterns were compared to the PulseNet USA database and named following the standardized PulseNet USA pattern naming scheme [16]. The electrophoresis was performed with a CHEF DR III System (Bio-Rad Laboratories, Hercules, CA, USA) using 1% SeaKem Gold agarose in 0.5× Tris-borate-EDTA. Running conditions consisted of increasing pulse times of 2.2 – 63.8 s for 20 h at 6 V/cm on a 120 deg. angle in 14°C TBE buffer.

### Multiple-locus variable-number tandem repeat analysis

MLVA was performed at the Centers for Disease Control and Prevention (CDC) in the United States of America by following the standardized PulseNet USA protocol for *Salmonella* serovar Enteritidis (Laboratory standard operating procedure for PulseNet MLVA of *Salmonella*s serovar Enteritis – Beckman Coulter 8000 platform. Accessed at: http://www.pulsenetinternational.org and Laboratory standard operating procedure for analysis of MLVA data of *Salmonella* serovar Enteritidis in BioNumerics – Beckman Coulter 8000 data. Accessed at: http://www.pulsenetinternational.org)

### Analysis of the composite data set

Analysis of PFGE data was performed at CDC. Comparisons were performed using Bionumerics software version 5.01 (Applied Maths, Sint-Martens-Latem, Belgium). The composite analysis was based on equal weighting of *Xba*I, *Bln*I and MLVA data and unweighted pair group method with arithmetic mean (UPGMA) clustering.

## Results

### Description of the data sets

**Figure 1:**
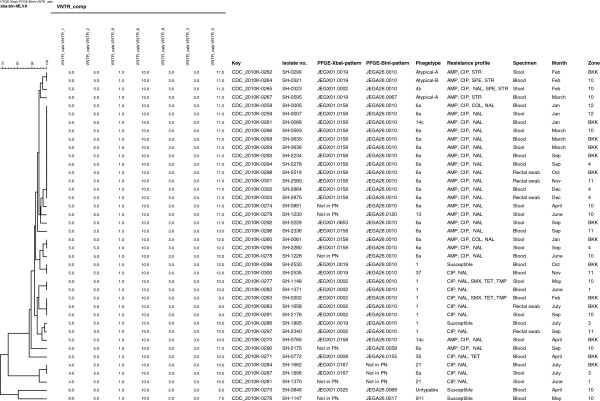
**A composite dendrogram based on PFGE and MLVA data containing 40 *Salmonella *** **serotype Enteritidis isolates from Thai patients.**

The 40 *Salmonella* serovar Enteritidis isolates selected for the analysis were all paired based on source of isolate. The pairs covered all months with exception of August and the geographical zones; BKK (n = 14), 1 (n = 2), 3 (n = 2), 4 (n = 4), 10 (n = 12), 11 (n = 4), and 12 (n = 2) (Figure 1).

### Antimicrobial resistance

The MIC determination of the 40 *Salmonella* serovar Enteritidis isolates revealed eight antimicrobial resistance profiles. The most common profile exhibited resistance to three antimicrobials: ampicillin, ciprofloxacin, and nalidixic acid. Nineteen (48%) and nine (23%) isolates belonged to the most common (AMP-CIP-NAL) and the second most common (CIP-NAL) resistance profiles, respectively (Table 1).

**Table 1:** **Frequency of the resistance profile per variable; specimen and geographical zone among *Salmonella enterica *** **serovar Enteritidis in Thai patients during 2008**

**Resistance profile**	**No of isolates**	**Specimen (No. (%))**		**Zone (No. (%))**						
		**Blood**	**Faeces**	**BKK**	**1**	**3**	**4**	**10**	**11**	**12**
AMP-CIP-NAL	19	8 (42)	11 (58)	7 (37)	0	0	4 (21)	5 (26)	2 (11)	1 (5)
CIP-NAL	9	3 (33)	6 (67)	2 (22)	2 (22)	1 (11)	0	2 (22)	2 (22)	0
CIP-NAL-SMX-TET-TMP	2	1 (50)	1 (50)	1 (50)	0	0	0	1 (50)	0	0
AMP-CIP-COL-NAL	2	1 (50)	1 (50)	1 (50)	0	0	0	0	0	1 (50)
AMP-CIP-STR	2	1 (50)	1 (50)	1 (50)	0	0	0	1 (50)	0	0
AMP-CIP-SPE-STR	1	1 (100)	0	0	0	0	0	1 (100)	0	0
CIP-NAL-TET	1	1 (100)	0	1 (100)	0	0	0	0	0	0
Pan-susceptible	4	4 (100)	0	1 (25)	0	1 (25)	0	2 (50)	0	0
Total	40	20 (50)	20 (50)	14 (35)	2 (5)	2 (5)	4 (10)	12 (30)	4 (10)	2 (5)

Abbreviations: AMP, ampicillin; CIP, ciprofloxacin; COL, colistin; NAL, nalidixic acid; SPT, spectinomycin; STR, streptomycin; SMX, sulfamethoxazole; TET, tetracycline; TMP, trimethoprim.

Ninety percent of the isolates (n = 36) were ciprofloxacin resistant (MIC 0.25 – 2 mg/L), and of these, 83% were also nalidixic acid resistant (MIC >64 mg/L). Seven percent of the isolates exhibited resistance to ciprofloxacin (MIC 1 mg/L) while susceptible to nalidixic acid (MIC 16 mg/L). Four strains (10%) were pansusceptible. Overall, antimicrobial resistance was observed to ampicillin (60%), tetracycline (8%), streptomycin (8%), colistin (5%), sulfamethoxazole (5%), trimethoprim (5%), and spectinomycin (3%) (Table 1).

The most common antimicrobial resistance profile (AMP-CIP-NAL), contained a mixture of stool 11/19 (58%) and blood 8/19 (42%) isolates. Profiles; AMP-CIP-NAL, CIP-NAL, CIP-NAL-SMX-TET-TMP, AMP-CIP-COL-NAL, AMP-CIP-STR contained both blood and stool isolates. However, profiles AMP-CIP-SPE-STR, CIP-NAL-TET, and pansuceptible were composed solely of blood isolates. No profiles were present in all seven geographic zones. However, profiles AMP-CIP-NAL and CIP-NAL were observed in five out of seven zones (Table 1).

### Phage typing

Among the 40 isolates, 11 different phage types were observed: 6a (n = 19), 1 (n = 8), 14c (n = 2), 21 (n = 2), 4b (n = 1), 13 (n = 1), 35 (n = 1), 37 (n = 1), 911 (n = 1), three atypical lytic patterns, and one untypable (Figure 1). Significant variation in phage susceptibility was observed. Susceptibility to 11 typing phages differentiated the two most common phage types (6a and 11). Phage types 21, 35, & 37 differed by their susceptibility to four to six of the typing phages.

### Pulsed-field gel electrophoresis typing

Seven different previously known *Xba*I PFGE patterns [JEGX01.0158 (n = 16), JEGX01.0002 (n = 7), JEGX01.0019 (n = 6), JEGX01.0167 (n = 2), JEGX01.0008 (n = 1), JEGX01.0325 (n = 1), JEGX01.0653 (n = 1)] were identified among the 40 isolates in addition to six patterns which were new to the PulseNet USA database. The isolates were further subtyped using a second enzyme, *Bln*I, which revealed seven different previously known *Bln*I PFGE patterns [JEGA26.0010 (n = 31), JEGA26.0017 (n = 1), JEGA26.0058 (n = 1), JEGA26.0067 (n = 1), JEGA26.0068 (n = 1), JEGA26.0120 (n = 1), JEGA26.0155 (n = 1)] and two additional patterns which were new to the PulseNet USA database. In total 14 *Xba*I/*Bln*I PFGE pattern combinations were detected (Figure 1).

### Multiple-locus variable-number tandem repeat analysis

The 40 strains generated seven different MLVA types. Variation was observed at loci VNTR-1 (n = 4), VNTR-2 (n = 2), VNTR-5 (n = 8) and VNTR-9 (n = 2). The most common profile (5-5-1-10-3-3-11) contained 20 isolates. (Figure 1). Three isolates displayed variation both at loci VNTR-1 and VNTR-5 (allelic profile: 4-5-1-10-3-3-10), one isolate displayed variation in three loci VNTR-1, VNTR-5 and VNTR-9 (allelic profile: 8-5-1-10-2-3-7), one isolated showed variation in four loci VNTR-1, VNTR-2, VNTR-5 and VNTR-9 (allelic profile: 6-6-1-10-2-3-6), and the remaining 15 isolates exhibited variation only at locus VNTR-5 (Figure 1).

### Analysis of the composite data set

Composite analysis based on PFGE and MLVA data grouped the 40 isolates into 22 genotypes. Seven genotypes contained multiple isolates; 15 genotypes were comprised of a single isolate. No single genotype was responsible for either gastroenteritis or bacteremia among Thai patients. In Five instances, the same genotype was isolated from both stool and blood in different zones and time periods (Figure 1).

## Discussion

Previous studies indicated that infection with *Salmonella* serovar Enteritidis was a statistically significant risk factor for bacteremia among Thai patients [7,17,18]. The goal of this study was to characterize *Salmonella* serovar Enteritidis isolates obtained from blood and stool specimens in Thailand in a spatial and temporal context and determine if a particular clone is associated with bacteremia based on the information described by Hendriksen *et al.*[7]. Isolates selected to insure geographic, seasonal, and age distribution. An equal number of stool and blood isolates were tested from each geographic zone. Patient logs were reviewed to insure that only one isolate per patient was tested.

This study utilized multiple subtyping methods as means to determine the relatedness of blood and stool isolates. A composite analysis based on PFGE and MLVA data revealed 22 unique genotypes among 40 isolates. Five genotypes consisting of at least two isolates contained an equal number of blood and stool isolates. All of the seven multi-isolate genotypes contained multiple phage types and/or antibiogrammes. These data indicate that multiple *Salmonella* serovar Enteritidis strains are circulating in the Thai population and that no specific clones were associated with a higher risk of bacteremia.

*Salmonella* serovar Enteritidis is typically regarded as a monophyletic serovar and the diversity observed among the isolates in this study is noteworthy [19]. This diversity may suggest that these strains originated from multiple reservoirs. Comparison of these strains to food, animal, and environmental isolates of *Salmonella* serovar Enteritidis in Thailand may lead to the identification of reservoirs and assist with the implementation of control measures [20]. Although non-human data is limited, the incidence of *Salmonella* serovar Enteritidis among Thai chickens dramatically increased from 1.17% in 1991 to 10.37% in 1992 [21]. The increase continued peaking in 1994 with 33.8% of frozen chicken meat being contaminated with *Salmonella* serovar Enteritidis [17] and then declined to 14.2% in 2002 [22]. Characterization of poultry isolates and comparison of these isolates to human Enteritidis isolates may provide additional insight into the epidemiology of this organism.

In a risk factor analysis performed on the top 10 *Salmonella* serovars reported in Thailand between 2002–2007, *Salmonella* serovars I 4,5,12:i:- and Typhimurium were also isolated from blood at an increased rate when compared to other NTS (28.6% and 28.2% respectively) [7]. Several studies have shown that immunocompromised individuals are at a significantly higher risk for the development of bacteremia due to *Salmonella* serovars Enteritidis or Typhimurium. A previous survey of bloodstream infections conducted in Northeastern Thailand between 1989 and 1998 indicated an increase in blood stream infections directly associated with HIV infection and caused by Group D non-typhoidal Salmonellae; primarily *Salmonella* serovar Enteritidis. [23]. Several studies from other countries in the region revealed similar epidemiology of *Salmonella* serovar Enteritidis associated with bacteremia in HIV patients [24-26].

The isolates characterized in previous studies were typically resistant to co-trimoxazole, likely due to its widespread use for *Pneumocystis jiroveci* prophylaxis in HIV positive patients [2,27-29]. Although we do not have information on the HIV status of the patients included in this study, it is interesting to note that only two out of 40 isolates characterized in this study were resistant to co-trimoxazole. Thus, it would be of value to ascertain the HIV status of the patients infected with *Salmonella* serovar Enteritidis in Thailand.

We observed limited antimicrobial resistance among the 40 *Salmonella* serovar Enteritidis isolates tested. This was in agreement with the general perception that *Salmonella* serovar Enteritidis is not a highly antimicrobial resistant serovar [30,31]. However, 83% of the tested isolates exhibited resistance to ciprofloxacin and nalidixic acid. Of note, 7% of the isolates exhibited resistance to ciprofloxacin and susceptibility to nalidixic acid. This phenotype may indicate possible plasmid-mediated quinolone resistance mechanism [32]. Quinolone resistance in *Salmonella* serovar Enteritidis has previously been described from Korea and Denmark and potential loss of this first line therapeutic is cause for concern. However, the reported data from Korea and Denmark were far from the high percentages described in this study with 90% resistance to ciprofloxacin [30,31]. The data in this study may indicate the presence of selection pressure from the use of fluoroquinolones. Such use within reservoirs for *Salmonella* serovar Enteritidis such as poultry, has previously been described [33]. This resistance is problematic as fluoroquinolones, which have been designated by the World Health Organisation as highly critical for human health, are often the main treatment for invasive salmonellosis in humans [31,33].

Phage types PT4, PT8, and PT 13 which are traditionally associated with poultry and cause the majority of human cases in the Western countries, were not identified [34,35]. Interestingly, uncommon phage types, primarily PT6a and PT1, were identified. Despite their “rarity”, these phage types have been previously identified in poultry from Thailand. In earlier reports, Phage type 4 was the most common *Salmonella* serovar Enteritidis phage type identified among human and poultry isolates (73.9%, n = 138 and chicken meat/feces; 76.2%, n = 164). However, PT1 and PT6a were also reported and accounted for 8.0%/3.7% and 0%/0.6% of the isolates recovered from humans and chickens respectively [36]. Also, as shown in previous studies from Korea and Denmark, *Salmonella* serovar Enteritidis PT1 appears to be previously associated with increased rates of nalidixic acid resistance. [30,31].

PFGE has typically provided limited discrimination for *Salmonella* serovar Enteritidis. However, the use of multiple restriction enzymes increases the discriminatory power of PFGE [19]. In this study, we used the enzymes *Xba*I and *Bln*I for the analysis and fairly diverse patterns were observed. These patterns are relatively rare and seldom reported to the US PulseNet database (CDC unpublished data) indicating, as for the previously mentioned methods, that the *Salmonella* serovar Enteritidis isolates in Thailand are distinct from strains circulating in North America. MLVA has recently emerged as a sequence-based alternative for PFGE and phage typing [37]. However, as in this study, it is best used as a complementary technique to other methods in order to reach a maximum discriminatory power for *Salmonella* serotype Enteritidis. The 7 patterns observed among the Thai isolates are all rare in the US PulseNet database (CDC, unpublished data) supporting the conclusions made based on PFGE and phage typing data.

## Conclusion

This study indicates that multiple subtypes of *Salmonella* serovar Enteritidis are circulating in Thailand and no single strain appears to be associated with a disproportionate number of blood stream infections. Previous studies have associated immunocomprimised conditions or malaria with an increased risk of bloodstream infections due to *Salmonella enterica* serovars Enteritidis and Typhimurium. Future efforts should focus on assessing the immune status of bacteriaemic patients and identifying prevention and control measures, including attribution studies characterizing non-clinical (animal, food, and environmental) isolates.

## Authors’ contributions

CP, SP, PC identified and serotyped all isolates as well as provided epidemiological data. RA carried out the phagetyping. CAS carried out the pulsed field gel electrophoresis. ES participated in the design of the study and performed the statistical analysis. EHT carried out the MLVA, the analysis, and helped to draft the manuscript. MM participated in design, the analysis, and helped to draft the manuscript. RSH conceived of the study, participated in its design, coordination, and draft the manuscript. All authors read and approved the final manuscript.
